# Clinical Application and Safety of Intrathecal Adjuvants in Single-Shot Thoracic Segmental Spinal Anesthesia: A Narrative Review

**DOI:** 10.7759/cureus.116360

**Published:** 2026-09-16

**Authors:** Imran Ahmed Khan, Naresh Wamanrao Paliwal, Harbhajan S Saini

**Affiliations:** 1 Anesthesiology, Neelkanth Hospital, Gorakhpur, IND; 2 Community Medicine, Keshav Memorial Charity (KMC) Medical College and Hospital, Maharajganj, IND; 3 Anesthesiology, Dr. Panjabrao Deshmukh Memorial Medical College, Amravati, IND; 4 Anesthesiology, Apollo Hospital, Jabalpur, IND

**Keywords:** adjuvant, dexmedetomidine, fentanyl, opioid, spinal anesthesia

## Abstract

Thoracic segmental spinal anesthesia (TSSA) is an emerging regional anesthesia (RA) technique. TSSA with local anesthetic (LA) alone is associated with a relatively short duration of surgical anesthesia and postoperative analgesia, which may limit its clinical utility. Intrathecal adjuvants have been widely used in lumbar spinal anesthesia (LSA) to prolong block duration and improve block quality. However, their role in TSSA remains poorly defined. This review summarizes commonly used intrathecal adjuvants during TSSA, their doses, adverse effects, and safety considerations.

A comprehensive literature search was performed using PubMed, Europe PubMed Central (PMC), and Google Scholar. Key search terms included “thoracic spinal anesthesia,” “thoracic segmental spinal anesthesia,” “segmental thoracic spinal anesthesia,” “intrathecal adjuvant,” “fentanyl,” “dexmedetomidine,” and “clonidine.” A total of 463 relevant articles were identified from PubMed (39), Europe PMC (26), and Google Scholar (398). Finally, 76 articles were selected for the review. An important observation from this review is the growing role of adjuvants in TSSA.

Common intrathecal adjuvants in TSSA include opioids and α2-agonists and, less commonly, benzodiazepines, corticosteroids, and N-methyl-D-aspartate (NMDA) antagonists. Several studies reported satisfactory surgical conditions with the preservation of spontaneous ventilation and improved hemodynamic stability. This finding has important implications for high-risk patients with severe cardiopulmonary diseases. Intrathecal adjuvants significantly improve block quality, prolong analgesia, and reduce local anesthetic requirements. Fentanyl and dexmedetomidine are the most extensively studied adjuvants in TSSA; morphine, buprenorphine, and ketamine have also been evaluated in a smaller number of studies and appear clinically useful. However, adverse effects such as hypotension, bradycardia, respiratory depression, and opioid-related complications remain important concerns.

## Introduction and background

Thoracic segmental spinal anesthesia (TSSA) is an emerging regional anesthesia (RA) technique that involves the administration of a small dose of local anesthetic (LA) into the thoracic subarachnoid space to achieve a selective block (segmental) at the required dermatomal level while minimizing the motor blockade of the lower limbs [1]. This technique has gained interest in surgeries involving the chest wall, breast, and abdomen because of its potential advantages, including hemodynamic stability, rapid recovery, and the avoidance of general anesthesia (GA) [2-4]. This aligns with the goals of enhanced recovery after surgery (ERAS) pathways.

The concept of TSSA is not a recent innovation. In 1909, Thomas Jonnesco published his work titled “General spinal analgesia” after intrathecal drug deposition in the thoracic region. Jonnesco proposed this technique for surgeries of the skull, head, neck, and thorax, performing punctures between the first and second thoracic vertebrae. For surgeries of the lower thoracic segment, he performed a puncture between the 12th thoracic vertebra and the first lumbar vertebra, which was more easily accomplished and produced anesthesia for the lower portion of the body [5]. Despite these early descriptions, the technique remained largely dormant for decades, primarily due to concerns about the risk of direct spinal cord injury. The contemporary era of TSSA began in 2006 when van Zundert et al. proposed segmental spinal block for laparoscopic cholecystectomy in a patient with severe lung disease, using a low thoracic puncture at T10 with a combined spinal-epidural technique [6].

However, TSSA with LA alone is often associated with a relatively short duration of surgical anesthesia and postoperative analgesia, which may limit its clinical utility [7]. Intrathecal adjuvants such as opioids (fentanyl and sufentanil), α2-agonists (dexmedetomidine and clonidine), and other agents (dexamethasone and magnesium) have been widely used in lumbar spinal anesthesia (LSA) to prolong block duration, improve block quality, and provide better postoperative analgesia [8-10]. These agents act synergistically at spinal receptors to produce their clinical effects. However, their role in TSSA remains poorly defined. Fentanyl and dexmedetomidine are the most extensively studied adjuvants in TSSA; morphine, buprenorphine, and ketamine have also been evaluated in a smaller number of studies and appear clinically useful.

The existing literature on TSSA is heterogeneous, with varying doses of LAs and adjuvants, different surgical populations, and inconsistent outcome reporting. Currently, no published review specifically synthesizes the evidence regarding the influence of intrathecal adjuvants on the clinical performance of TSSA. Therefore, a literature review is necessary to critically appraise the available evidence, identify knowledge gaps, and guide future clinical practice and research. This review summarizes the commonly used intrathecal adjuvants during TSSA, their doses, adverse effects, and safety considerations.

## Review

Methods

A comprehensive literature search was performed using PubMed/Medical Literature Analysis and Retrieval System Online (MEDLINE), Europe PubMed Central (PMC), and Google Scholar. Key search terms included “thoracic spinal anesthesia,” “thoracic segmental spinal anesthesia,” “segmental thoracic spinal anesthesia,” “intrathecal adjuvant,” “fentanyl,” “morphine,” “dexmedetomidine,” and “clonidine.”

English-language, comparative, observational studies; randomized controlled trials (RCTs); case reports; and case series were included. Adult patients > 18 years of age in whom TSSA was used as an intrathecal adjuvant in combination with one of the local anesthetic drugs (levobupivacaine, bupivacaine, and ropivacaine) were included. We investigated both Food and Drug Administration (FDA)-approved and off-label agents, as only morphine, ziconotide, and baclofen are approved for intrathecal use.

TSSA combined with any other block, including epidurals and conference abstracts without full data, was excluded. Studies in which TSSA was performed but outcomes of interest were not reported, pediatric or obstetric populations, studies using adjuvants administered via non-intrathecal routes, and continuous spinal, review articles, editorials, correspondence, letters to the editor, perspectives, and opinion papers without data were excluded.

Two independent reviewers (IAK and HSS) screened titles/abstracts, followed by full-text assessment using Rayyan software (Rayyan Systems Inc., Cambridge, MA). Disagreements were resolved by consulting with the third (NWP) reviewer.

Results and discussion

A total of 463 potentially relevant articles were identified from PubMed (39), Europe PMC (26), and Google Scholar (398). After removing 42 records marked as ineligible by automation tools and 64 duplicate articles, 357 were available for title and abstract screening. Screening of titles and abstracts further removed 242 articles. Of the remaining 115, the full texts of 13 articles were not retrieved and were removed. The full text of the remaining 102 articles was retrieved and assessed, and based on the selection criteria, 26 articles were excluded at this stage. Finally, 76 articles were selected for the review presented in Figure 1 [11-87].

**Figure 1:**
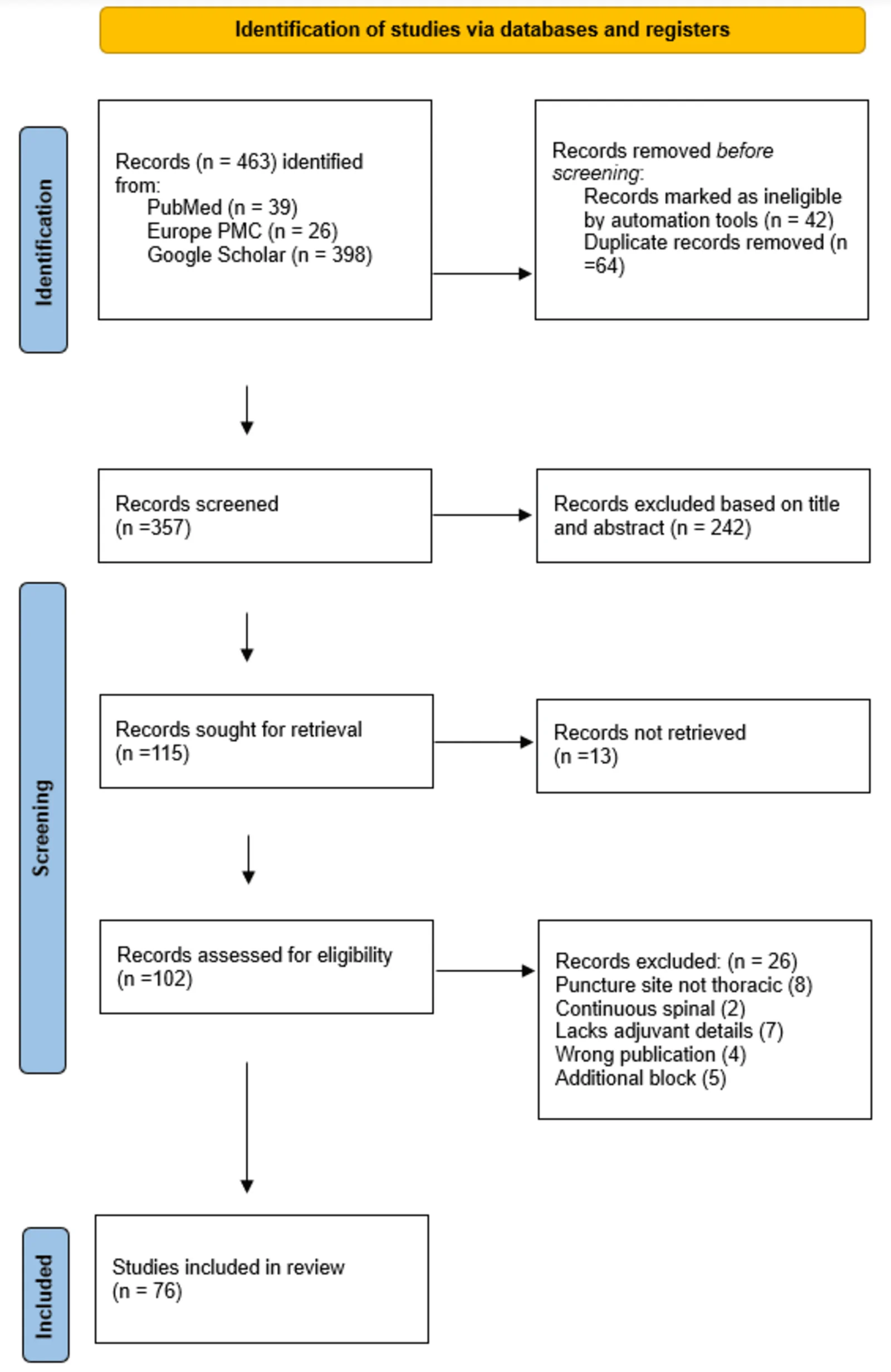
PRISMA flow diagram of selected articles PRISMA, Preferred Reporting Items for Systematic Reviews and Meta-Analyses; PMC, PubMed Central

Current Evidence and Clinical Applications of Intrathecal Adjuvants

The re-emergence of TSSA coincides with increasing interest in low-dose neuraxial techniques, awake surgery, and opioid-sparing perioperative care. Although traditional LSA has long incorporated intrathecal adjuvants, their role in TSSA has recently received broader attention. The principal rationale for adding adjuvants to TSSA includes improving block quality, prolonging postoperative analgesia, reducing LA dose requirements, improving visceral pain control, enhancing patient comfort, maintaining hemodynamic stability, attenuating excessive sympathetic blockade, and providing intrathecal sedation in some protocols [33,88,89]. TSSA is primarily utilized as an alternative to GA or LSA for surgical procedures involving the thorax and upper abdomen, particularly in high-risk or elderly patients [20,22,71].

Commonly Used Intrathecal Adjuvants in TSSA

These additives are generally categorized into different classes based on their pharmacological mechanisms (Table 1). Common intrathecal adjuvants in TSSA include opioids and α2-agonists and, less commonly, benzodiazepines, corticosteroids, N-methyl-D-aspartate (NMDA) antagonists, and vasopressors.

**Table 1:** Intrathecal adjuvants in TSSA TSSA, thoracic segmental spinal anesthesia; NMDA, N-methyl-D-aspartate

Class of adjuvants	Agent with doses	Primary benefit	References
Opioids	Sufentanil (2.5 μg)	Enhanced analgesia and dose reduction of local anesthetics	[11]
	Fentanyl (15-25 μg)		[12-18,20-25,28-32,34-36,40-45,48-50,52,54-58,60,64,67,69-72,74,78,79,81-83,86,87]
	Morphine (100-300 μg)		[65,77,85]
	Buprenorphine (30-120 μg)		[39,53]
Alpha-2 agonists	Dexmedetomidine (5-10 μg)	Prolonged block, hemodynamic stability, and sedation	[19,26,27,36-38,40,41,43,47,48,51,56,61-63,66,68,69,75,76,80,87]
	Clonidine (10-20 μg)		[59]
Benzodiazepines	Midazolam (0.03 mg/kg, 1-2 mg)	Improved block quality and intrathecal sedation	[46,84]
Corticosteroids	Dexamethasone (4-6 mg)	Extended sensory block duration	[67,69]
NMDA antagonists	Ketamine (0.3 mg/kg, 5-20 mg)	Prevention of central sensitization and prolonged duration	[33,36,40,41,84]

Opioids

The most established class is intrathecal opioids, especially fentanyl, sufentanil, morphine, and buprenorphine. Fentanyl and sufentanil are lipophilic opioids, while morphine is a hydrophilic opioid. Other less commonly reported opioids include butorphanol and nalbuphine [90,91]. Butorphanol and nalbuphine were not reported in the TSSA in the included studies.

Opioid adjuvants continue to play an important role in neuraxial anesthesia. Fentanyl remains highly effective for visceral pain control and intraoperative comfort during laparoscopic and abdominal procedures. Morphine, although associated with prolonged analgesia, carries a higher risk of delayed respiratory depression and other opioid-related adverse effects.

Fentanyl: Intrathecal fentanyl consistently improves visceral analgesia and intraoperative comfort, particularly during laparoscopic procedures. The faster onset of sensory block and improved patient satisfaction have been reported in multiple studies. Adverse effects associated with fentanyl include pruritus, postoperative nausea and vomiting, and occasional urinary retention. It is the most frequently reported adjuvant in TSSA, administered at doses of 15-25 μg.

Morphine: Intrathecal morphine provided prolonged postoperative analgesia that extended into the first postoperative day. However, delayed respiratory depression, nausea, pruritus, and urinary retention are significant concerns.

Buprenorphine: Buprenorphine, an opioid adjuvant, has been used intrathecally to improve the quality of the block and provide extended postoperative analgesia during TSSA. Clinical reports indicate that the use of buprenorphine in TSSA is associated with stable hemodynamics, with no instances of hypotension, bradycardia, or respiratory depression in documented case series [39,53].

Alpha-2 Adrenergic Agonists

The second major class is α2-adrenergic agonists, mainly dexmedetomidine and clonidine. These adjuvants prolong the block effect for an extended time.

Dexmedetomidine: Dexmedetomidine was the most frequently studied non-opioid intrathecal adjuvant in TSSA. Most studies demonstrated prolonged sensory blockade, delayed requirement for rescue analgesia, and improved patient comfort when dexmedetomidine was added to low-dose bupivacaine. The sympatholytic properties of dexmedetomidine may also contribute to improved perioperative stress modulation. However, higher incidences of bradycardia and hypotension have been observed in some patient populations, particularly elderly individuals. Nevertheless, caution is warranted in elderly and hemodynamically vulnerable patients because of the increased risk of bradycardia and hypotension.

Clonidine: Clonidine demonstrated prolonged postoperative analgesia and reduced postoperative opioid requirement. However, prolonged sedation and hypotension limit its use in ambulatory and short-duration surgeries.

NMDA Antagonists/Inorganic Chemicals

These adjuvants have been reported in a few studies. Their routine use will require further high-level evidence.

Magnesium sulfate: Limited evidence suggests that magnesium sulfate may prolong analgesia through N-methyl-D-aspartate receptor antagonism. However, a delayed onset of sensory blockade and inconsistent analgesic benefits have been reported [92,93]. No direct evidence of magnesium sulfate as an adjuvant was found in the TSSA literature in included studies.

Ketamine: In addition, the NMDA antagonist ketamine has been used intrathecally to prolong block duration and, in some protocols, to provide intrathecal sedation when combined with other agents.

Benzodiazepines

Midazolam: It is used intrathecally to enhance the quality of the block and provide sedation. In some major surgeries, it is combined with agents such as ketamine to provide intrathecal sedation with stable hemodynamics.

Glucocorticoids

Dexamethasone: Dexamethasone is used in TSSA as a glucocorticoid adjuvant to enhance the quality of the block and provide extended analgesia. It is a key component in “opioid-free” TSSA protocols, helping to achieve effective pain control without the side effects associated with intrathecal opioids.

Sympathomimetics

Adrenaline (epinephrine): Although less commonly used now, it can prolong the block duration through local vasoconstriction and increase the intensity of the motor block [94]. This has not been explored in TSSA.

Clinical effects of intrathecal adjuvants

An important observation from this review is the growing role of adjuvants in ultra-low-dose TSSA. Several studies reported satisfactory surgical conditions with the preservation of spontaneous ventilation and improved hemodynamic stability. This has important implications for high-risk patients with severe cardiopulmonary disease who may not tolerate GA.

This review also highlights substantial heterogeneity in the literature. Variability in thoracic puncture levels, local anesthetic regimens, adjuvant doses, surgical procedures, and outcome definitions limited direct comparison across studies. Furthermore, most studies were small and single-centered, thereby reducing external validity.

Hemodynamic Outcomes

This review demonstrates that intrathecal adjuvants may significantly enhance the efficacy and clinical applicability of TSSA. The use of adjuvants appears particularly valuable in achieving adequate anesthesia with reduced doses of LAs, thereby minimizing excessive sympathetic blockade and hemodynamic instability. Several studies have highlighted the role of adjuvants in facilitating ultralow-dose spinal anesthesia with reduced sympathetic blockade. TSSA combined with low-dose LA and adjuvants was associated with improved hemodynamic stability compared to conventional LSA.

R*espiratory Outcomes*

Most studies reported the preservation of spontaneous ventilation and minimal respiratory compromise during awake TSSA. However, respiratory depression remains a concern with intrathecal opioid adjuvants.

Patient Satisfaction

Improved patient satisfaction was commonly reported owing to prolonged postoperative analgesia, reduced systemic opioid requirements, and the avoidance of GA.

Safety Considerations

Intrathecal adjuvants have an established role in TSSA to improve block characteristics and prolong postoperative analgesia. However, certain safety concerns should be considered when using intrathecal adjuvants to optimize beneficial effects during TSSA. The potential adverse effects are listed in Table 2. Common concerns include delayed respiratory depression, itching, sedation, and urinary retention. The long-term neurological safety of intrathecal adjuvants remains a concern. Although current evidence suggests acceptable short-term safety with preservative-free formulations, robust long-term neurotoxicity data are limited for some agents.

**Table 2:** Safety concerns with intrathecal adjuvants

Clinical concerns	Adjuvant class	References
Pruritus	Fentanyl and morphine	[11,13,15,70]
Sedation	Buprenorphine, dexmedetomidine, and ketamine	[19,26,37,38,41,59,83]
Bradycardia and hypotension	Dexmedetomidine and clonidine	[16,17,26,37,50,51,76]
Respiratory depression	Fentanyl and morphine	[44,58]
Urinary retention	Fentanyl and morphine	[44,58]

Current Challenges and Controversies

Despite growing enthusiasm surrounding intrathecal adjuvants in TSSA, several unresolved issues remain. Most intrathecal adjuvants are currently used off-label [95]. Substantial heterogeneity exists in thoracic puncture levels, the baricity of LAs, adjuvant doses, and outcome definitions across available studies. This variability complicates the interpretation of the literature and limits direct comparisons between studies. Long-term neurological safety data remain limited for several adjuvants, particularly when used intrathecally in TSSA. Although preservative-free formulations appear relatively safe, concerns regarding neurotoxicity persist. Another important controversy involves the balance between prolonged analgesia and delayed recovery. Although agents such as dexmedetomidine and morphine improve postoperative analgesia, they may also contribute to hypotension, bradycardia, sedation, urinary retention, and delayed ambulation [96-98].

Moreover, the role of opioid-free TSSA remains incompletely defined. Although α2-adrenergic agonists may reduce perioperative opioid requirements, robust comparative data evaluating opioid-free and opioid-based thoracic neuraxial strategies are lacking. The increasing interest in awake surgery, ERAS pathways, and opioid-sparing perioperative care will likely continue to drive research into intrathecal adjuvants for TSSA. Future studies should focus on standardized protocols, comparative effectiveness analyses, dose optimization, and patient-centered recovery outcomes.

This review is subject to several limitations. The existing literature on intrathecal adjuvants in TSSA is highly heterogeneous, with considerable variability in thoracic puncture levels, LA regimens, and adjuvant doses, which precludes direct comparisons. Furthermore, the majority of included studies are low-level evidence, thereby limiting the external validity and generalizability of the findings. The widespread off-label use of most intrathecal adjuvants also raises regulatory and safety concerns, and the lack of robust long-term neurotoxicity data for several agents underscores the need for further high-quality research. There are high chances of selection bias owing to the review type. Limited database searches may skip some relevant articles.

## Conclusions

Intrathecal adjuvants enhance the scope and effectiveness of TSSA by improving block quality, prolonging analgesia, and reducing LA requirements. Fentanyl and dexmedetomidine appear to be the most extensively studied and clinically useful agents in current TSSA practice. Morphine, buprenorphine, and ketamine have also been evaluated in a smaller number of studies and appear clinically useful. However, adverse effects such as hypotension, bradycardia, respiratory depression, and opioid-related complications remain important concerns. Despite promising results, the existing evidence remains heterogeneous and limited by small sample sizes and the lack of standardized protocols. For patient-specific decisions, further high-quality research is essential to establish optimal adjuvant selection, dosing strategies, and the long-term safety of TSSA.
